# Supplementary material for: Structural insight into ligand binding and activation of the orphan GPCR Mas1
Source: EMBO J. 2026 Mar 30;45(10):3500–13. doi: 10.1038/s44318-026-00764-6 (PMC13187490; doi:10.1038/s44318-026-00764-6)
Supplement: Supplementary file 1 — Appendix [file 44318_2026_764_MOESM1_ESM.pdf]

Appendix for Structural Insight into Ligand Binding and Activation of the GPCR Orphan Receptor Mas1

**Appendix Figure S1**	**Page 2**
**Appendix Figure S2**	**Page 3-4**
**Appendix Figure S3**	**Page 5-6**
**Appendix Figure S4**	**Page 7**
**Appendix Figure S5**	**Page 8**
**Appendix Figure S6**	**Page 9**
**Appendix Figure S7**	**Page 9**
**Appendix Figure S8**	**Page 10**
**Appendix Table S1**	**Page 11**
**Appendix Table S2**	**Page 12**
**Appendix Table S3**	**Page 13-14**
**Appendix Table S4**	**Page 14**

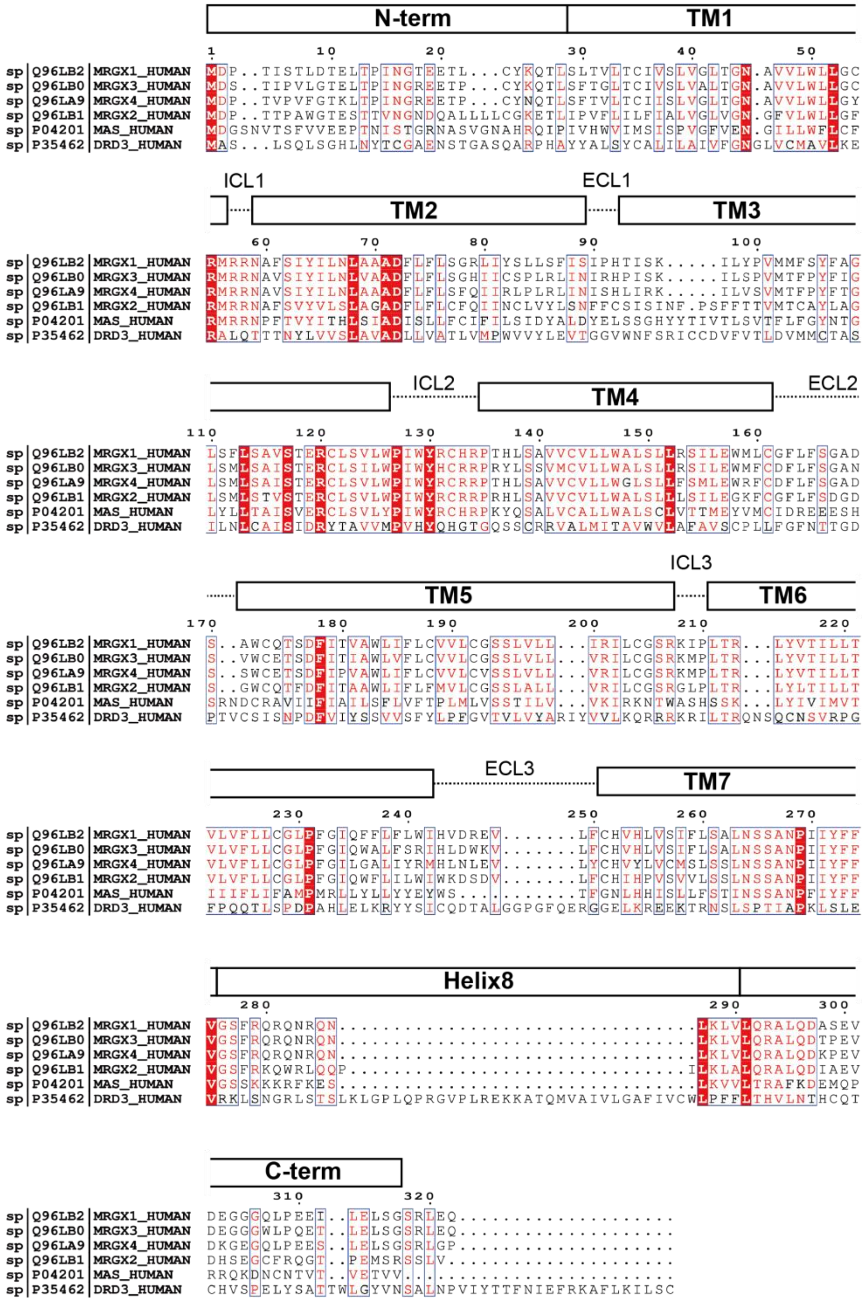

Appendix Fig S1 The sequences alignment and the conservation of the interaction sites between Mas1, DRD3 and MRGPRX family receptors. a, Sequence alignment of the Mas1, DRD3 and MRGPRX family receptors created by CLUSTALW (https://www.genome.jp/tools-bin/clustalw) and ESPript 3.0 (https://espript.ibcp.fr/ESPript/cgi-bin/ESPript.cgi).

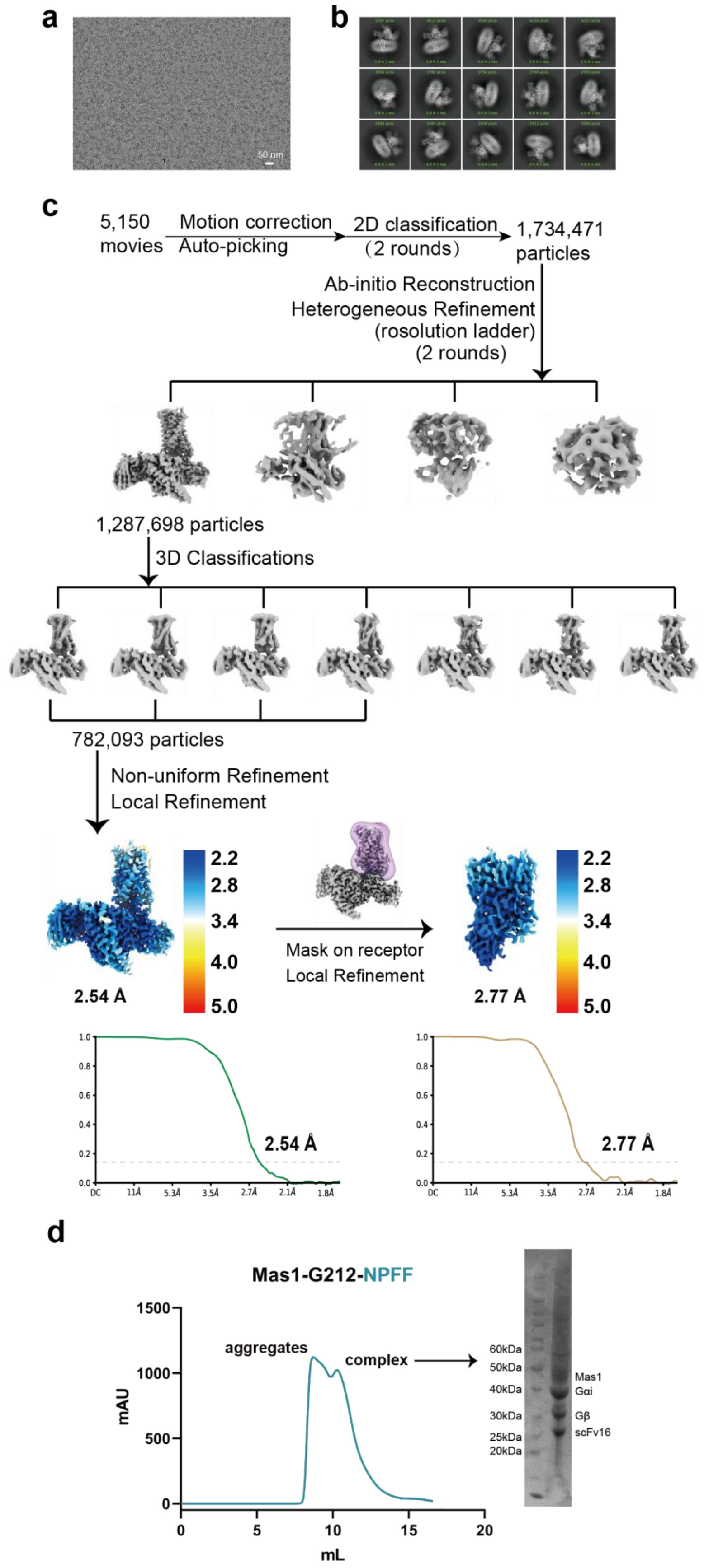
a
50 nm
b
c
5,150 movies
Motion correction
Auto-picking
2D classification
(2 rounds)
1,734,471 particles
Ab-initio Reconstruction
Heterogeneous Refinement
(rosolution ladder)
(2 rounds)
1,287,698 particles
3D Classifications
782,093 particles
Non-uniform Refinement
Local Refinement
2.2
2.8
3.4
4.0
5.0
2.54 Å
Mask on receptor
Local Refinement
2.77 Å
2.54 Å
2.77 Å
d
Mas1-G212-NPFF
aggregates
complex
1500
1000
500
0
mAU
0
5
10
15
20
mL
60kDa
50kDa
40kDa
30kDa
25kDa
20kDa
Mas1
Gαi
Gβ
scFv16

Appendix Fig S2 NPFF-Mas1-G_i complex purification and cryo-EM data processing a, Representative cryo-EM micrograph of vitrified NPFF–Mas1–G_i particles embedded in vitreous ice. Scale bar, 50 nm. b, Selected two-dimensional (2D) class averages from cryo-EM data processing, showing various particle views and orientations. c, Computational sorting of cryo-EM particle images, representative 3D reconstructions from heterogeneous refinement, the “Gold-standard” FSC curve. d, Representative size-exclusion chromatography elution profile and SDS-PAGE analysis.

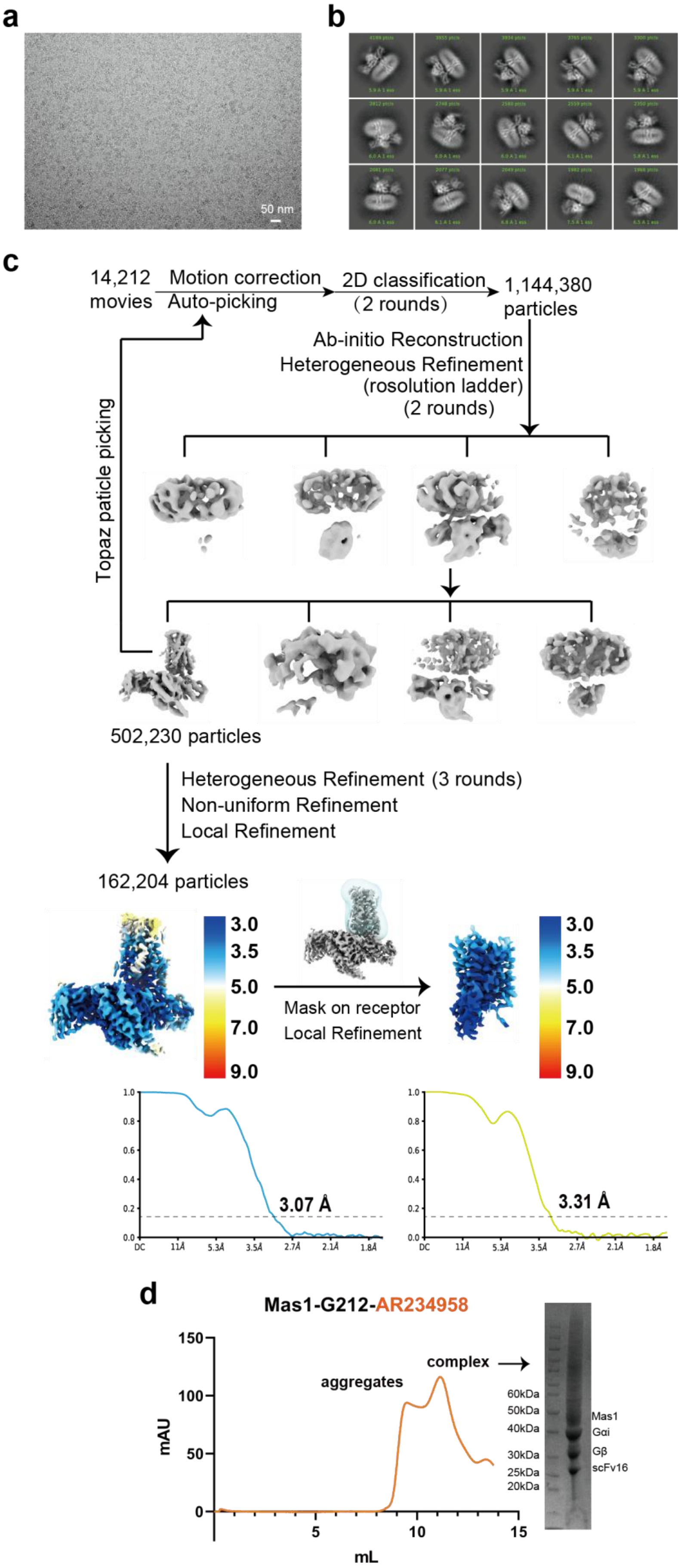
a
50 nm
b
c
14,212 movies
Motion correction
Auto-picking
2D classification
(2 rounds)
1,144,380 particles
Ab-initio Reconstruction
Heterogeneous Refinement
(rosolution ladder)
(2 rounds)
Topaz paticle picking
502,230 particles
Heterogeneous Refinement (3 rounds)
Non-uniform Refinement
Local Refinement
162,204 particles
3.0
3.5
5.0
7.0
9.0
Mask on receptor
Local Refinement
3.0
3.5
5.0
7.0
9.0
3.07 Å
3.31 Å
DC
11Å
5.3Å
3.5Å
2.7Å
2.1Å
1.8Å
d
Mas1-G212-AR234958
complex
aggregates
mAU
mL
150
100
50
0
5
10
15
60kDa
50kDa
40kDa
30kDa
25kDa
20kDa
Mas1
Gαi
Gβ
scFv16

Appendix Fig S3 AR234958-Mas1-Gi complex purification and cryo-EM data processing a, Representative cryo-EM micrograph of vitrified AR234958–Mas1–Gi particles embedded in vitreous ice. Scale bar, 50 nm. b, Selected two-dimensional (2D) class averages from cryo-EM data processing, showing various particle views and orientations. c, Computational sorting of cryo-EM particle images, representative 3D reconstructions from heterogeneous refinement, the “Gold-standard” FSC curve. d, Representative size-exclusion chromatography elution profile and SDS-PAGE analysis.

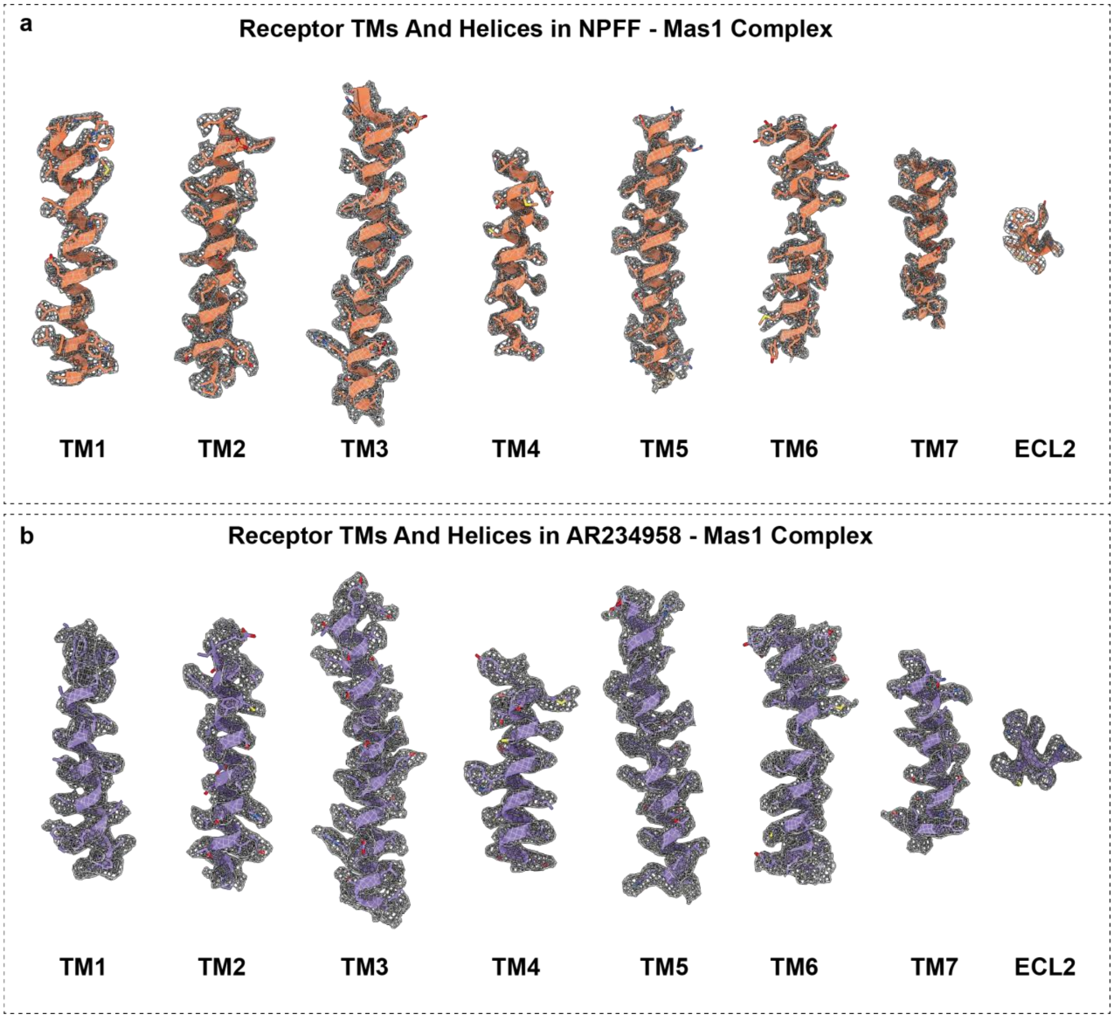

Appendix Fig S4 Representative cryo-EM density maps of the TM helices in the NPFF–Mas1–G_i and AR234958–Mas1–G_i complexes a, Cryo-EM density maps of the seven transmembrane (TM) helices of Mas1 in the Gi-bound NPFF–Mas1 complex, shown with the fitted atomic model. b, Corresponding cryo-EM density maps for the Gi-bound AR234958–Mas1 complex. All TM helices exhibit well-defined densities, including ECL2.

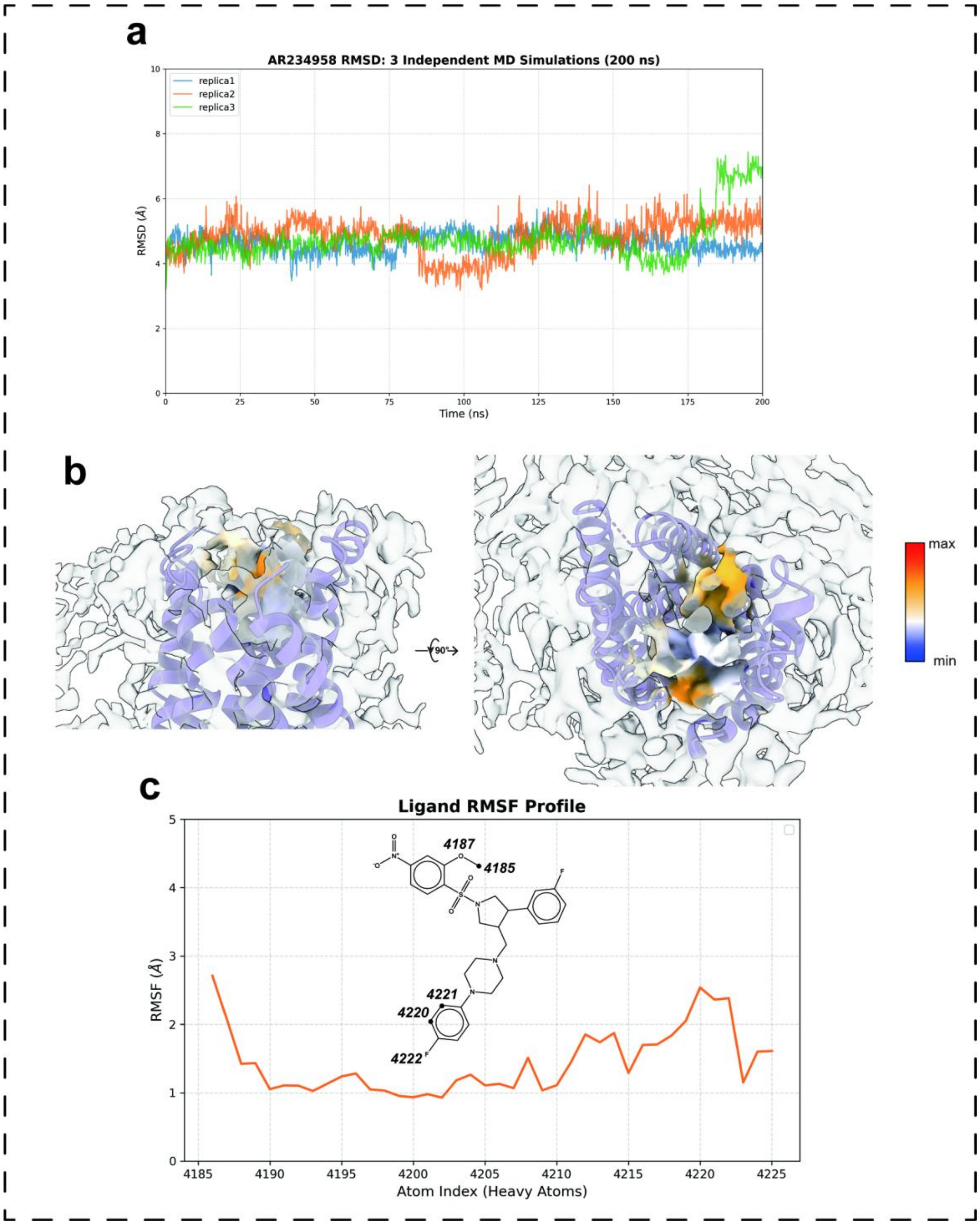

Appendix Figure S5: Molecular Dynamics Simulations Reveal the Conformational Flexibility of AR234958 Binding. (a) Root-Mean-Square Deviation (RMSD) plot of ligand heavy atoms from three independent 200-ns MD simulations (Replica 1–3). All trajectories show stable binding. (b) Structural mapping of ligand flexibility onto the cryo-EM density map. The ligand is colored by per-atom RMSF values (blue: low flexibility; Red: high flexibility) calculated from the representative trajectory (Replica 1). High-RMSF regions, particularly the peripheral aromatic rings, correspond to areas with weaker electron density (transparent gray surface), explaining the local map quality. (c) Per-atom Root-Mean-Square Fluctuation (RMSF) profile of AR234958 heavy atoms. Inset: 2D chemical structure of AR234958 with key flexible atoms labeled, corresponding to the high-RMSF peaks in the plot. The terminal phenyl rings exhibit significant mobility (> 2.0 Å), consistent with the structural observations in (b).

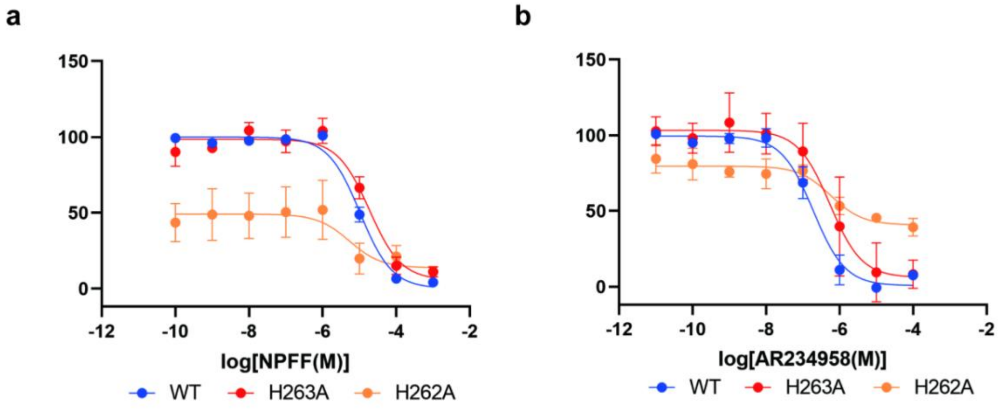

Appendix Fig S6 Functional effects of H262A and H263A mutations on MAS1-mediated inhibition of cAMP accumulation. Dose-response curves showing cAMP accumulation in HEK293 cells expressing MAS1 wild-type (WT, blue), H263A mutant (red), or H262A mutant (orange) receptors upon stimulation with either NPFF (a) or AR234958 (b). Cells were pre-treated with forskolin to induce cAMP production, followed by increasing concentrations of the indicated ligands. cAMP levels were normalized to maximal inhibition induced by NPFF (left) or AR234958 (right), and data are expressed as percentage of maximal response. Each point represents mean ± SEM of the three independent experiments performed in duplicate. The H262A mutation markedly reduced ligand-induced cAMP inhibition, suggesting impaired Gαi coupling, whereas H263A retained partial activity.

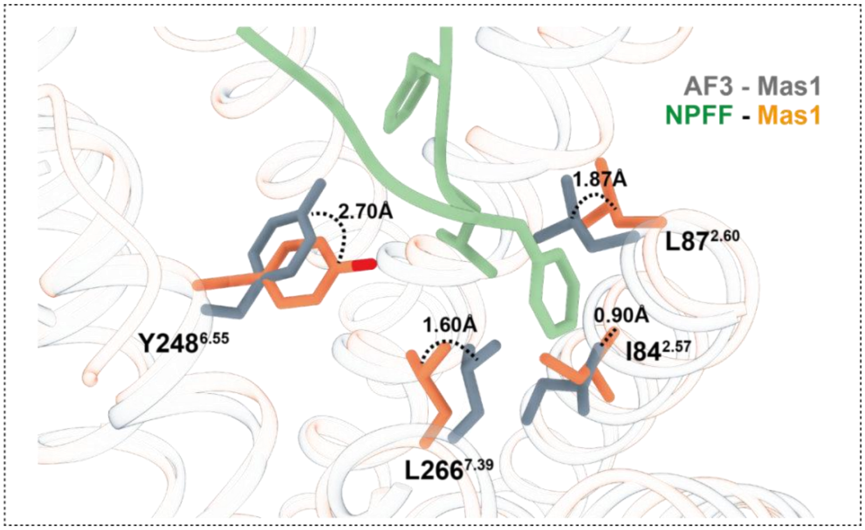

Appendix Fig S7 Structural comparison of AF3 predicted Mas1 and NPFF–bound Mas1 complexes reveals the compression at the ligand-binding pocket. Superimposed views of MAS1 receptor structures bound to NPFF (orange) and AF3 (gray), with NPFF shown in green stick representation. Notable structural rearrangements include inward shifts of I84$^{2.57}$ (0.90 Å), L87$^{2.60}$ (1.87 Å), and L266$^{7.39}$ (1.60 Å). The side chain of Y248$^{6.55}$ also undergoes a displacement of 2.70 Å toward the ligand pocket. These conformational shifts highlight a compression of the orthosteric binding pocket in the NPFF–MAS1 complex, revealing a ligand-specific binding mode characterized by a compaction mechanism.

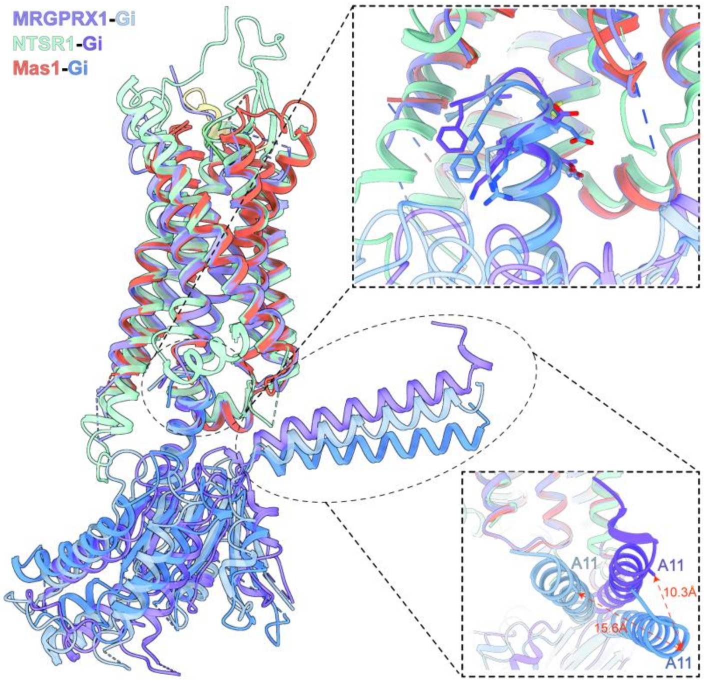

Appendix Fig S8 Cross-receptor comparison of Gi-coupled GPCR complexes reveals divergent Gαi engagement Superimposed views of MRGPRX1–Gi (purple/blue), NTSR1–Gi (cyan/green), and MAS1–Gi (salmon/red) aligned by the receptor seven-transmembrane (7TM) core. The top-right inset compares the orthosteric pockets, with the MRGPRX1 ligand shown as dark-blue sticks. The central dashed ellipse highlights differences in the position and tilt of the Gαi α5 helix relative to the receptor core. The bottom-right inset quantifies the lateral shift of the Gαi αN helix, measured at Ala11 (A11) Cα, yielding representative pairwise separations of 10.3 Å and 15.6 Å.

Appendix Table S1 pEC_{50} values of three ligands on WT Mas1. The cAMP accumulation assay indirectly evaluates Gi protein activation by measuring the suppression of forskolin-stimulated cAMP production. Data are presented as means ± S.E.M. of three independent experiments (n=3), each consisting of triplicate measurements. The dataset links to Fig. 1.

**Effects of Wild Type (Mas1) on Ligands-mediated inhibition of forskolin-induced cAMP accumulation**		
**Ligand**	**pEC_{50} ± S.E.M. (%WT）**	**Span ± S.E.M. (%WT)**
AR234958	6.713 ± 0.116	98.895 ± 4.722
NPFF	4.982 ± 0.063	99.890 ± 2.955
Ang1-7	3.131 ± 0.103	99.452 ± 5.610

Appendix Table S2 pEC_{50} values of NPFF on Mas1 mutants. The cAMP accumulation assay indirectly evaluates Gi protein activation by measuring the suppression of forskolin-stimulated cAMP production. Data are presented as means ± S.E.M. of three independent experiments (n=3), each consisting of triplicate measurements. All data were analyzed by two-side, one-way ANOVA with Dunnett's test. *P<0.05, **P<0.01, ***P<0.001 vs. wild-type (WT). The dataset links to Fig. 3e, f, g.

**Effects of mutation on NPFF-mediated inhibition of forskolin-induced cAMP accumulation**					
**Mutant**	**pEC_{50} ± S.E.M. （%WT）**	**P value**	**Span ± S.E.M. （%WT）**	**P value**	**Surface Expression (%WT) ± S.E.M.**
WT	4.982 ± 0.063	/	99.890 ± 2.955	/	100 ± 1.774
H36A	5.334 ± 0.266	0.9907	61.928 ± 6.956	0.0716	99.394 ± 2.145
I39A	4.616 ± 0.250	0.9885	62.591 ± 7.305	0.0791	93.263 ± 1.018
I84A	N.D	/	N.D	/	94.550 ± 0.330
L87A	4.651 ± 0.294	0.9926	68.175 ± 9.359	0.1741	68.206 ± 1.791
Y91A	N.D	/	N.D	/	94.777 ± 1.130
S109A	N.D	/	N.D	/	89.629 ± 1.213
Y116A	4.635 ± 0.393	0.9914	86.923 ± 15.937	0.9355	91.181 ± 2.567
L120A	4.970 ± 0.250	＞ 0.9999	67.224 ± 7.871	0.1531	83.535 ± 2.455
Y168A	N.D	/	N.D	/	61.669 ± 2.514
I172A	4.581 ± 0.493	0.9780	51.763 ± 11.935 *	0.0142	98.789 ± 3.358
F237A	N.D	/	N.D	/	59.019 ± 1.942
A241G	4.864 ± 0.260	0.9997	92.465 ± 11.317	0.9969	92.127± 1.772
M244A	N.D	/	N.D	/	93.111 ± 1.775
Y248A	N.D	/	N.D	/	86.374 ± 1.140
H262A	5.274 ± 0.715	0.9966	25.859 ± 5.217 ***	0.0009	89.553 ± 1.313
H263A	4.732 ± 0.156	0.9975	92.860 ± 6.757	0.9972	96.694 ± 3.059

L266A	4.081 ± 0.365	0.4304	40.608 ± 8.542 **	0.0021	109.273 ± 0.657

Appendix Table S3. pEC_{50} values of the small molecular AR234958 on Mas1 mutants. The cAMP accumulation assay indirectly evaluates Gi protein activation by measuring the suppression of forskolin-stimulated cAMP production. Data are presented as means ± S.E.M. of three independent experiments (n=3), each consisting of triplicate measurements. All data were analyzed by two-side, one-way ANOVA with Dunnett's test. **P*＜0.05，***P*＜0.01，****P*＜0.001 vs. wild-type (WT). The dataset links to Fig. 4e, f, g.

**Effects of mutation on AR234958-mediated inhibition of forskolin-induced cAMP accumulation**					
**Mutant**	**pEC50 ± S.E.M. （%WT）**	**P value**	**Span ± S.E.M. （%WT）**	**P value**	**Surface Expression (%WT) ± S.E.M.**
WT	6.713 ± 0.116	/	98.895 ± 4.722	/	100 ± 1.774
I84A	N.D	/	N.D	/	94.55 ± 0.330
L87A	N.D	/	N.D	/	68.206 ± 1.791
Y91A	N.D	/	N.D	/	94.777 ± 1.130
Y95A	6.669 ± 0.262	0.9999	64.875 ± 6.966	0.0765	113.891 ± 1.196
Y168A	N.D	/	N.D	/	61.669 ± 2.514
I172A	5.566 ± 0.392	0.1185	29.246 ± 5.368 ***	0.0003	98.789 ± 3.358
H262A	6.365 ± 0.614	0.9557	38.275 ± 9.830 **	0.0011	89.553 ± 1.313
H263A	6.265 ± 0.337	0.8711	97.029 ± 14.118	0.9998	96.694 ± 3.059
L266A	N.D	/	N.D	/	109.273 ± 0.657

Appendix Table S4 Cryo-EM data collection, refinement and validation statistics.

	Mas1-Gi complex			
	**NPFF**	**Mas1-local**	**AR234958**	**Mas1-local**
Voltage (kV)	300		300	
Electron exposure (e^-/Å^2)	50		50	
Defocus range (μm)	-1.0 to -2.0		-1.0 to -2.0	
Pixel size (Å)	0.83		0.83	
Symmetry imposed	C1		C1	
Filtered particle images (no.)	1,734,471		1,114,380	
Final particle images (no.)	782,093		162,204	
Map resolution (Å)	2.54	2.77	3.07	3.31
FSC threshold	0.143	0.143	0.143	0.143
Map resolution range (Å)	2.2 - 5.0	2.2 - 5.0	3.0 - 9.0	3.0 - 9.0
**Refinement**				
Initial model used (PDB code)	AlphaFold Mas1	AlphaFold Mas1	AlphaFold Mas1	AlphaFold Mas1
Map sharpening *B* factor (Å^2)	-100.8	-152.8	-119.3	-171.6
Model composition				
Non-hydrogen atoms	8,360	2,015	8,343	2,012
Protein residues	1,073	247	1,066	242
Ligands	--	--	1	1
B factors (Å^2)				
Protein	96.14	61.11	150.20	150.20
Ligand	--	--	115.26	115.26
R.m.s. deviations				
Bond lengths (Å)	0.004	0.004	0.005	0.004
Bond angles (°)	0.658	0.862	0.966	0.954
Validation				
MolProbity score	1.20	1.39	1.47	1.59
Clashscore	2.66	3.44	4.30	4.70
Poor rotamers (%)	0.00	0.00	0.00	0.00
Ramachandran plot				
Favored (%)	97.23	96.23	96.16	94.92
Allowed (%)	2.77	3.77	3.84	5.08
Disallowed (%)	0.00	0.00	0.00	0.00
