# Supplementary figures and images for: Structural insight into ligand binding and activation of the orphan GPCR Mas1
Source: EMBO J. 2026 Mar 30;45(10):3500–13. doi: 10.1038/s44318-026-00764-6 (PMC13187490; doi:10.1038/s44318-026-00764-6)

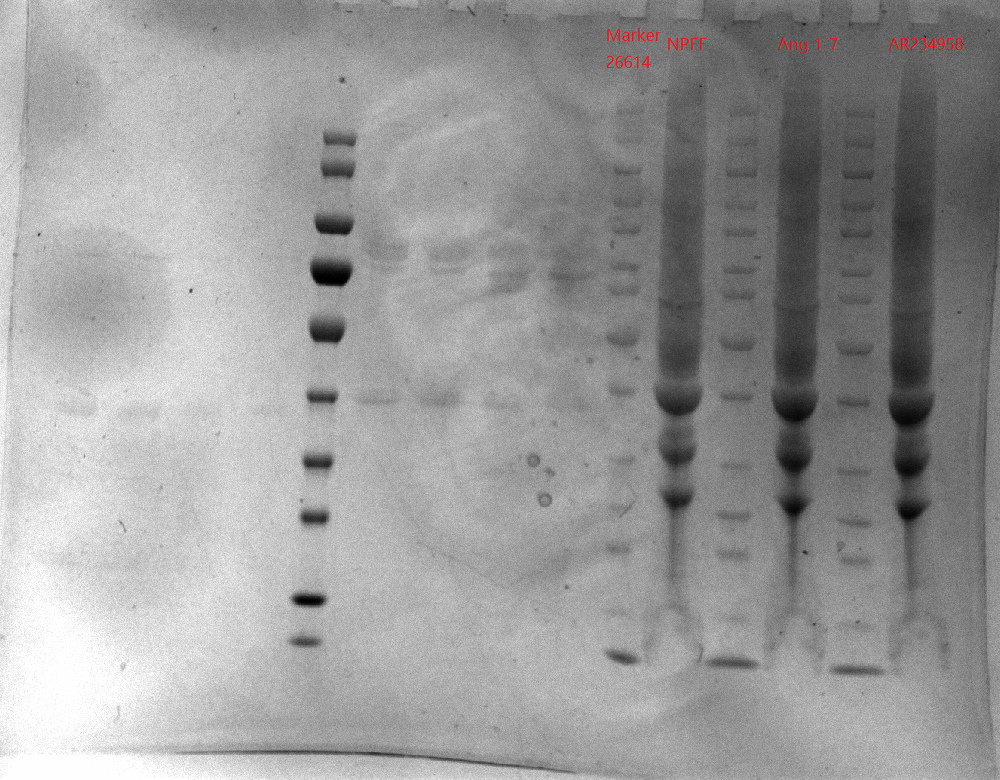

Supplement: Supplementary file 7 — Appendix Figure Source Data [file 44318_2026_764_MOESM7_ESM.zip › Appendix Source Data/Sup.figure_2/Sup.figure 1/original gel scn file by BioRad.png]
